# Identification and characterization of deschloro-chlorothricin obtained from a large natural product library targeting aurora A kinase in multiple myeloma

**DOI:** 10.1007/s10637-020-01012-2

**Published:** 2020-09-25

**Authors:** Nadire Özenver, Sara Abdelfatah, Anette Klinger, Edmond Fleischer, Thomas Efferth

**Affiliations:** 1 Department of Pharmacognosy, Faculty of Pharmacy, Hacettepe University, 06100 Ankara, Turkey; 2 Department of Pharmaceutical Biology, Institute of Pharmaceutical and Biomedical Sciences, Johannes Gutenberg University, Staudinger Weg 5, 55128 Mainz, Germany; 3 MicroCombiChem GmbH, 65203 Wiesbaden, Germany

**Keywords:** Cancer, Multiple myeloma, Natural product, precision medicine, Targeted chemotherapy

## Abstract

Multiple myeloma (MM) is a devastating disease with low survival rates worldwide. The mean lifetime of patients may be extendable with new drug alternatives. Aurora A kinase (AURKA) is crucial in oncogenesis, because its overexpression or amplification may incline the development of various types of cancer, including MM. Therefore, inhibitors of AURKA are innovative and promising targets. Natural compounds always represented a valuable resource for anticancer drug development. In the present study, based on virtual drug screening of more than 48,000 natural compounds, the antibiotic deschloro-chlorotricin (DCCT) has been identified to bind to AURKA with even higher binding affinity (free bindung energy: −12.25 kcal/mol) than the known AURKA inhibitor, alisertib (free binding energy: −11.25 kcal/mol). The in silico studies have been verified in vitro by using microscale thermophoresis. DCCT inhibited MM cell lines (KMS-11, L-363, RPMI-8226, MOLP-8, OPM-2, NCI-H929) with IC_50_ values in a range from 0.01 to 0.12 μM. Furthermore, DCCT downregulated AURKA protein expression, induced G2/M cell cycle arrest and disturbed the cellular microtubule network as determined by Western blotting, flow cytometry, and fluorescence microscopy. Thus, DCCT may be a promising lead structure for further derivatization and the development of specific AURKA inhibitors in MM therapy.

## Introduction

Multiple myeloma (MM) is a critical plasma cell proliferative disorder characterized by the accumulation of malignant plasma cells in the bone marrow, bone lesions and immunodeficiency causing 1% of all cancers, and 10% of hematological malignancies [1–3]. Based on the Surveillance, Epidemiology, and End Results (SEER) Program data from 2010 to 2016 published by National Cancer Institute (NCI), 5-year relative survival of patients with myeloma is 53.9% [4], which may be increased by newly developed drugs. Multi-drug resistance (MDR), a phenomenon where tumors gain resistance towards structurally and functionally diverse drugs at the same time, is a substantial obstacle leading to treatment failure in cancer therapy, including MM [5, 6]. Therefore, novel treatment regimens targeting innovative proteins or signaling pathways involved in drug resistance may have the potential to overcome drug resistance and to extend patients’ survival time for individuals with MM.

Aurora kinases (AURKs) including aurora kinases A, B, and C are members of the serine/threonine kinase family and are key players involved in genetic stability during cell division [7]. They have significant roles in oncogenesis, *i.e.* their overexpression or amplification are involved in tumorigenesis of lung cancer, colorectal carcinoma, and melanoma. Moreover, AURK inhibitors (AKIs) prohibiting the occurrence of radio- and chemo-resistance are innovative and promising drug candidates in cancer [8, 9]. However, only a small number of AKIs have been investigated in clinical trials due to their frequently high toxicity towards healthy cells [10, 11]. In this regard, nature as a unique source for novel chemical scaffolds may represent a valuable repository of selective and effective compounds with few side effects. To exemplify, 113 out of 136 new chemical entities (83%) excluding non-biologicals and vaccines in the field of anticancer drugs were either natural products, natural product derivatives or natural product mimics in the time frame covered from 1981 to 2014 [12]. Therefore, natural compounds/products may provide ample opportunities for the discovery of novel drug leads, which may enable the design of prospective (semi)synthetic derivatives with improved pharmacological features such as AKIs.

Aurora A kinase (AURKA) is a centrosomal kinase with fundamental roles during mitosis including centrosome maturation, nuclear envelope breakdown, mitotic entry, centrosome separation, spindle pole formation, and spindle checkpoint activity [13, 14]. Overexpression of AURKA has been announced in numerous cancer types, such as laryngeal, ovarian, breast cancers [15]. Furthermore, the association of AURKA with cell proliferation, epithelial-mesenchymal transition, metastasis, chemoresistance, and self-renewal capacity of cancer stem cells have been previously reviewed [9, 16].

MM is characterized by a genetic imbalance with various chromosomal deformities [17]. AURKA sets in centrosomes in the early S phase and during mitosis an AURKA fraction correlates with spindle microtubules proximal to the spindle poles [3]. AURKA has a role in cytokinesis and is activated by phosphorylation during G2/M phase transition in the cell cycle. Impaired AURKA may incline aneuploidy characteristics of tumors [3, 18]. AURKA upregulation is associated with centrosome amplification and worse prognosis in MM [19–21]. Inhibition of AURKA expression in MM cells inclined apoptosis through abrogation of G2/M cell cycle progression [20, 21]. Taken together, inhibition of AURKA may be a convincing strategy in MM therapy.

In the present research, we screened over 48,000 compounds from a natural product library of the ZINC database. We aimed to discover natural drug candidates as AURKA inhibitors in MM by the combined use of both in silico and in vitro approaches. Furthermore, the elucidation of modes of action of identified candidate molecules in MM cells was also planned. Within the context of the present research, our findings may stimulate the derivatization of lead compounds for the development of prospective (semi)synthetic compounds or combination regimens in MM targeting AURKA.

## Materials and methods

### Cell lines

MM cell lines comprising KMS-11, MOLP-8, NCI-H929, L-363, RPMI-8226, OPM-2 were originated from the Leibniz Institute DSMZ-German Collection of Microorganisms and Cell Cultures. The cells were provided by Dr. Ellen Leich (University of Würzburg) and Dr. Manik, Chatterjee (University of Würzburg) [22].

The cell lines were cultured in RPMI 1640 medium, supplemented with 10% fetal bovine serum (FBS) (Invitrogen) and 1% penicillin (100 U/ml)-streptomycin (100 μg/ml) (PIS) antibiotic (Invitrogen) and incubated in humidified 5% CO_2_ atmosphere at 37 °C. Cells were passaged twice weekly. All experiments were conducted with the cells in their logarithmic growth phase.

U2OS human osteosarcoma cancer cells, stably transfected with an α-tubulin-GFP construct, were obtained from Dr. Joachim Hehl (Light Microscopy Centre, ETH Zürich, Switzerland). The cells were cultured in DMEM medium with 10% FBS and, 1% penicillin (100 U/ml)-streptomycin (100 μg/ml) (PIS) antibiotic (Invitrogen) and continuously treated with 250 μg/ml geneticin at 37 °C and 5% CO_2_ to maintain α-tubulin expression.

### Chemical and reagents

A library of 9 compounds was provided by MicroCombiChem GmbH (Wiesbaden, Germany).

### Virtual screening

ZINC15 contains over 48,000 purchasable secondary metabolites from natural origin, the data of which were obtained from the ZINC database (https://zinc.docking.org) [23]. The crystal structure of AURKA was downloaded from the Protein Data Bank (https://www.rcsb.org) [24] as a PDB file (PDB code: 4UYN) [25]. 20 amino acids of AURKA (Gly 140, Lys 141, Val 147, Ala 160, Lys 162, Leu 164, Leu 178, Val 182, Gln 185, Leu 194, Leu 208, Leu 210, Glu 211, Tyr 212, Ala 213, Gly 216, Leu 263, Ala 273, Asp 274, Phe 275) were taken into consideration to form the grid box for defined docking [25]. A structurally based virtual screening was then conducted using PyRx software (http://pyrx.scripps.edu).

Parts of this research were carried out using the supercomputer Mogon and advisory services offered by Johannes Gutenberg Universiy Mainz (hpc.uni-mainz.de), which is a member of the Alliance for High Performance Computing in Rhineland-Palatinate (www.ahrp.info) and the Gauss Alliance e.V.

### Molecular docking with AutoDock 1.5.6

If virtual screening results with a binding affinity of ≤ −10.2 kcal/mol were achieved, the corresponding compounds were further taken to the molecular docking with AutoDock 4.2.6 (The Scripps Research Institute, CA, USA) [26]. AutoDockTools 1.5.6 was performed to prepare the files for the molecular docking. Protein and ligand files were converted to PDBQT (Protein Data Bank Partial Charge and Atom Type) files. The grid boxes were created as mentioned above in the virtual screening section around the amino acids of AURKA involved in ligand binding according to the literature [25].

Lamarckian Algorithm, calculating 250 runs and 25,000,000 energy evaluations for each cycle, was used in docking analysis, as previously decribed [27]. Docking log (dlg) files, containing the lowest binding, the mean binding affinity and the predicted inhibition constant (*pKi*), provided essential knowledge about docking results. Interacting amino acids (via hydrogen bonding and hydrophobic interactions) with the AURKA were identified using AutoDockTools. Visual Molecular Dynamics 1.9.3 (VMD) was used for docking visualizations (http://www.ks.uiuc.edu/Research/vmd/) [28]. As in the virtual screening, parts of this work were conducted using the supercomputer Mogon and advisory services offered by Johannes Gutenberg University Mainz (hpc.uni-mainz.de).

Based on the virtual screening and molecular docking results, 9 identified candidate compounds were provided by MicroCombiChem GmbH (Wiesbaden, Germany) for in vitro verification of the in silico results.

### Cytotoxicity assay

The cytotoxicity of the tested compounds has been studied by use of resazurin reduction assay [29, 30]. The assay depends on the reduction of resazurin to resarufin by viable cells. Non-viable cells do not exhibit a blue staining due to losing their metabolic capacity. Briefly, 1 × 10^4^ cells in a total volume of 100 μL were seeded in 96-well cell culture plate. The cells were incubated with various concentrations of the regarding compound to get a total volume of 200 μL/well for 72 h. Then, 0.01% of resazurin (Sigma-Aldrich, Germany) diluted in double-distilled water (ddH_2_O) was added (20 μL/well) and incubated for another 4 h. Infinite M2000 ProTM plate reader (Tecan, Germany) was used to measure the fluorescence using an excitation wavelength of 544 nm and an emission wavelength of 590 nm. Each assay was independently performed thrice with six parallel replicates each. Dose response curves of each cell were formed using GraphPad Prism® v6.0 software (GraphPad Software Inc., San Diego, CA, USA). The 50% inhibition concentrations (IC_50_) were calculated by nonlinear regression using Microsoft Excel.

### Microscale thermophoresis (MST)

Microscale thermophoresis (MST) was performed for assessment of the interaction between compound (5) and AURKA (Sigma-Aldrich, Germany). The method is performed as previously described [31, 32]. The AURKA protein was labeled using Monolith Protein Labeling Kit RED- NHS 2nd Generation (MO-L011, NanoTemper Technologies GmbH, Munich, Germany) according to manufacturer’s instructions. The AURKA protein concentration used was 400 nm, it was titrated against different concentrations of compound (5). Analysis buffer used includes; 50 mM Tris buffer pH 7.0, 150 mM NaCl, 10 mM MgCl_2_ and 0.05% Tween 20. Samples of interaction were filled into Monolith NT.115 standard capillaries (MO-K022, NanoTemper Technologies GmbH, Munich, Germany). Monolith NT.115 instrument (NanoTemper Technologies) was used for fluorescent signal measurement. Test was performed using 60% LED power and 40 MST power. For analysis, we used MO.Affinity analysis software (Nano Temper Technologies) to generate of fitting curve of interaction and calculation of dissociation constant (Kd).

### Analysis of cell cycle distribution by flow cytometry

The MOLP-8 cells (1 × 10^6^ cells/well) were seeded into 6-well plates and treated with ranging concentrations (0.5 × IC_50_, IC_50_, 2 × IC_50_ and 4 × IC_50_) of compound (5) for 24 h. The cells were collected, washed with phosphate buffered saline (PBS) and fixed with 96% ice-cold ethanol. Following the fixation of the cells, they were washed with PBS again, dissolved in PBS and stained with propidium iodide (PI, Sigma-Aldrich) at a final concentration of 50 μg/mL for 15 min at room temperature in the dark. The BD Accuri™ C6 Flow cytometer (Becton-Dickinson, Heidelberg, Germany) was used to perform cell cycle analyses at 488 nm excitation wavelength, and emission was measured by a 610/20 nm band pass filter. A total number of 1 × 10^4^ cells were counted for each experiment. All experiments were performed at least thrice [33].

### Imaging of structure and dynamics of the microtubule cytoskeleton by fluorescence misroscopy

U2OS-GFP -α-tubulin cells (5 × 10^5^/well) were seeded into 6-well plates, each including a sterile ibi Treat μ-slide (ibidi, Germany). The cells were enabled to attach overnight, treated with 10 or 25 μM of compound (5) or DMSO (solvent control) and incubated at 37 °C for 24 h. Then, the cells were rinsed with PBS and fixed by 4% *p*-formaldehyde at room temperatuere for 30 min. Subsequently, the cells were washed with PBS and stained for 5 min with 1 μM of 4′,6-diamidino-2-phenylindole (DAPI) (Life Technologies, Darmstadt, Germany), followed by washing with PBS again and mounting. Fluorescence imaging was performed by using 470 nm excitation and 525 nm emission for GFP and 447 nm emission for DAPI of EVOS digital inverted microscope (Life Technologies). Each experiment was done at least thrice and representative images were selected [34].

### Protein extraction

MOLP-8 cells (3 × 10^6^) were seeded in six-well plates and treated with various concentrations (0.01, 0.5, 1, 5, 10 μM) of compound (5) for 24 h. The cells were then washed with PBS and transferred into 1.5 ml Eppendorf tubes. M-PER® Mammalian Protein Extraction Reagent (Thermo Fisher Scientific, Germany) with protease inhibitor (1:100) was used for protein purification by shaking for 30 min at 4 °C. Then, cell lysates were centrifuged at 14,000×*g* for 15 min at 4 °C and the supernatants were shifted to the clean tubes. Total protein concentrations were calculated using a NanoDrop 1000 spectrophotometer (Thermo Scientific) [35].

### SDS PAGE and Western blot analysis

Thirty mg/mL of the protein fraction was taken and 5% β-mercaptoethanol including SDS-loading dye was added following by heating at 95 °C for 10 min. Subsequent to the denaturation process, the proteins were loaded onto 10% SDS-polyacrylamide gels. Then, a western blotting apparatus was used to transfer the proteins on a PVDF membrane (Roti® PVDF, pore size 0.45 μm, Carl Roth GmbH, Karlsruhe, Germany) Then, the membrane was blocked with blocking buffer (5% BSA in Tris-buffered saline Tween 20 (TBST)) for 1 h at room temperature and then incubated with primary antibody [Aurora A/AIK antibody rabbit polyclonal antibody (1:1000, Cell Signaling Technology, Frankfurt, Germany)] overnight at 4 °C. Then, HRP-linked anti-rabbit IgG (1:2000, Cell Signaling) was incubated with the membranes for 1 h. Luminata Classico HRP Western Blot substrate (Merck Millipore, Schwalbach, Germany) was used for the detection step and membranes were visualized by using Alpha Innotech FluorChem Q system (Biozym, Oldendorf, Germany) [32, 36].

## Results

### Virtual screening and molecular docking

Using more than 48,000 natural compounds from the ZINC database, we identified the top 105 compounds with a binding affinity equal to or less than −10.2 kcal/mol by virtual screening. Subsequently, defined docking calculations of these 105 compounds were performed by covering the residues involved in hydrogen bonds and hydrophobic interactions of AURKA and its selective inhibitor SAR156497 in the literature [25]. Based on the molecular docking results, the top 9 compounds unraveled higher binding affinities than that of alisertib (MLN8237, Milennium Pharmaceuticals, Inc., Cambridge, MA), which is a highly selective AURKA small-molecule inhibitor developed for the treatment of malignancies [37, 38] (Table 1). All 9 compounds bound to the same pharmacophore as SAR156497 and alisertib in defined docking approach and their binding affinities were ranged from −14.41 to −11.53 kcal/mol. Remarkably, all 9 compounds bound with higher affinity to AURKA than alisertib (Table 1). Four of them displayed only hydrophobic interactions, whereas the other five compounds formed both hydrogen bonds and hydrophobic interactions. In the context of in silico assessments, we elicited these 9 compounds for in vitro verification of computational results.

**Table 1:** PyRx and molecular docking results of alisertib and the top nine compounds with the highest binding affinity to AURKA in ZINC15 database

Numbered compounds	Compounds with ZINC ID	PyRx binding affinity (kcal/mol)	Binding affinity (kcal/mol)	Mean binding affinity (kcal/mol)	Predicted inhibition constant, pKi (nM)	H Bonds	Number of hydrophobic interactions
Compound 1	ZINC000079216661	−10.3	−14.41	−12.31	0.027	Lys 162	18
Compound 2	ZINC000118913887	−10.2	−12.68	−11.72	0.508	–	16
Compound 3	ZINC000004150075	−10.3	−12.61	−12.22	0.567	Ala 213	19
Compound 4	ZINC000077263184	−10.4	−12.43	−11.65	0.772	Lys 162	16
Compound 5	ZINC000252515584	−11.1	−12.25	−12.16	1.04	Arg 137 Val 174 Leu 178	15
Compound 6	ZINC000077262838	−10.3	−11.93	−11.88	1.79	–	16
Compound 7	ZINC000012530134	−10.7	−11.64	−11.40	2.93	–	16
Compound 8	ZINC000004236880	−10.4	−11.61	−11.10	3.10	–	12
Compound 9	ZINC000252495685	−10.4	−11.53	−10.76	3.55	Lys 162	14
–	Alisertib	–	−11.25	−10.07	5.67	Arg 137 Tyr 212	13

### Cytotoxicity

**Fig. 1:**
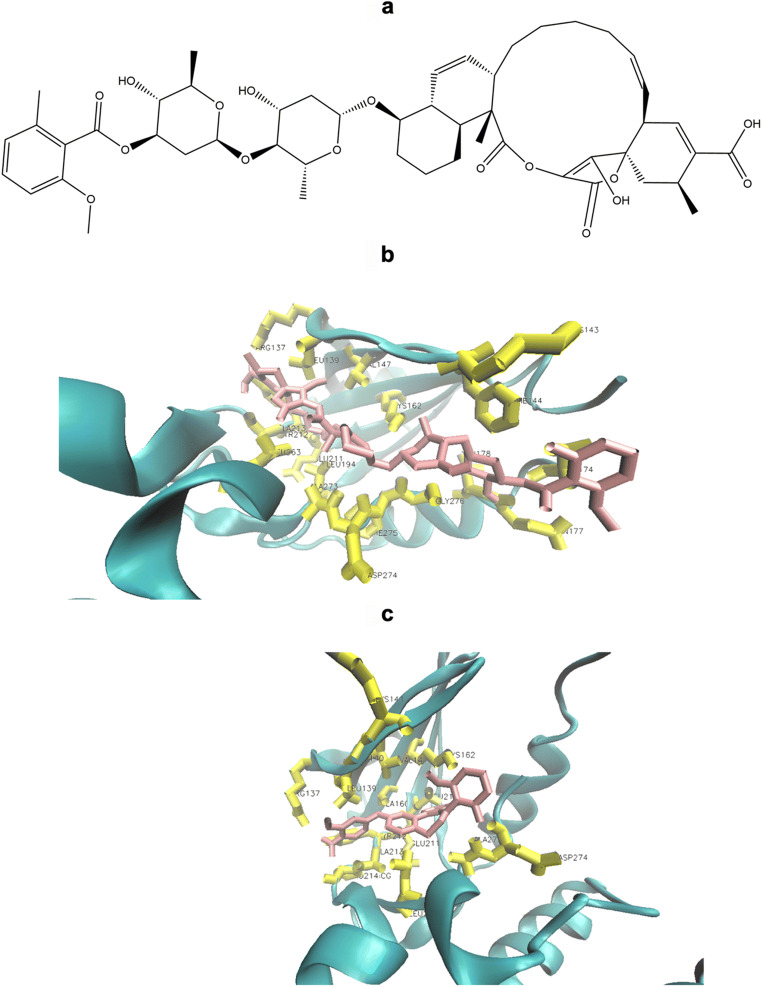
**a** The chemical structure of the compound (5) (deschloro-chlorothricin). **b** Molecular docking of deschloro-chlorothricin. Docking of deschloro-chlorothricin (pink) to the AURKA binding site (PDB code 4UYN). Deschloro-chlorothricin interacted with the amino acids (yellow) in the binding pocket. **c** Molecular docking of alisertib. Docking of alisertib (pink) to the AURKA binding site (PDB code 4UYN). Visual Molecular Dynamics 1.9.3 (VMD) was used for docking visualizations

**Fig. 2:**
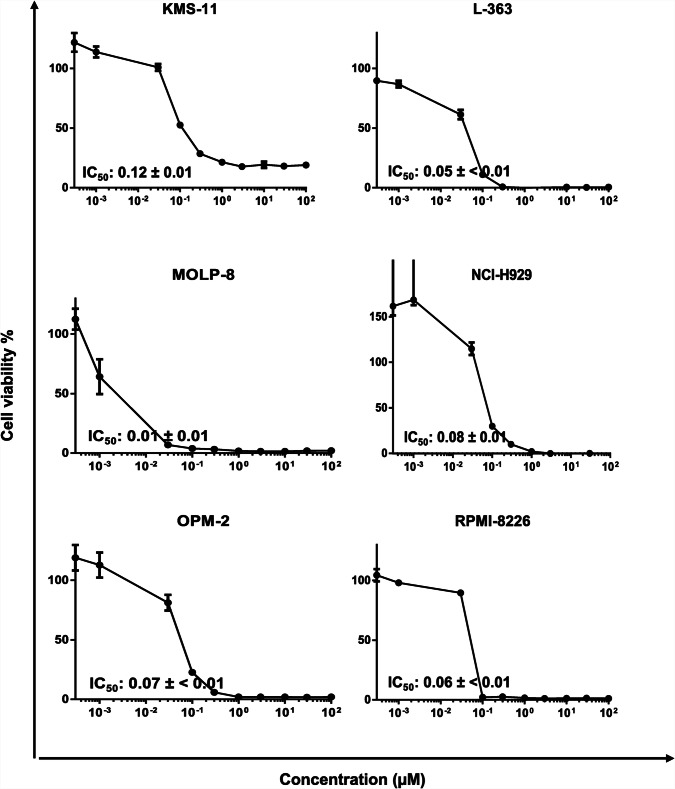
Dose-response curves of deschloro-chlorothricin. Cytotoxicity of deschloro-chlorothricin toward various multiple myeloma cells as determined by the resazurin assay. The results represent the mean ± SD of three independent experiments with six parallel measurements each

Preliminary screening was performed to determine cytotoxic drug candidates among the top 9 compounds by the evaluation of growth inhibition of various MM cells at a fixed concentration of 10 μM of each test compound. Although the cytotoxic responses of ZINC000252515584 (compound 5, a secondary metabolite termed “deschloro-chlorothricin” (DCCT) isolated from *Streptomyces antibioticus*. [23, 39]) and ZINC000077262838 were remarkable on numerous MM cells at a fixed concentration of 10 μM (Table 2), we focused only on ZINC000252515584 (deschloro-chlorothricin, DCCT) (Fig. 1) within the context of our research and further took it to the comprehensive cytotoxicity studies at ranging concentrations on more MM cells, because it was the only compound interacting with AURKA based on the microscale thermophoresis. DCCT was tested in numerous MM cells and dose-response curves were created (Fig. 2). It demonstrated 50% cell viability inhibition in multiple myeloma cells KMS-11, L-363, MOLP-8, NCI-H929, OPM-2 and RPMI-8226 at concentrations of 0.12 ± 0.01 μM, 0.05 ± < 0.01 μM, 0.01 ± 0.01 μM, 0.08 ± 0.01 μM, 0.07 ± < 0.01 μM, and 0.06 ± < 0.01 μM, respectively. Generally, these results indicated that MM cells were quite sensitive to DCCT. Particularly, MOLP-8 cells were the most sensitive cells among the other studied MM cells and were futher investigated to assess the mode of action of DCCT in these cells.

**Table 2:** Cytotoxicity screening of the top nine compounds based on the docking scores on three multiple myeloma cells including KMS-11, MOLP-8 and OPM-2 at 10 μM

Numbered compounds	Compounds	KMS-11	MOLP-8	OPM-2
		Cell viability at 10 μM	Cell viability at 10 μM	Cell viability at 10 μM
Compound 1	ZINC000079216661	98.22 ± 3.83	89.81 ± 5.12	96.32 ± 8.19
Compound 2	ZINC000118913887	41.65 ± 4.80	83.73 ± 4.83	74.97 ± 6.05
Compound 3	ZINC000004150075	91.08 ± 5.26	83.02 ± 6.65	78.05 ± 3.90
Compound 4	ZINC000077263184	88.88 ± 4.40	87.58 ± 1.00	84.60 ± 2.43
Compound 5	ZINC000252515584	14.87 ± 0.97	0.25 ± 0.07	2.98 ± 0.17
Compound 6	ZINC000077262838	23.26 ± 1.55	46.45 ± 4.19	14.91 ± 1.71
Compound 7	ZINC000012530134	45.55 ± 3.65	80.67 ± 4.88	76.56 ± 5.59
Compound 8	ZINC000004236880	88.21 ± 8.77	94.85 ± 2.03	80.49 ± 3.81
Compound 9	ZINC000252495685	83.44 ± 5.36	88.39 ± 4.33	84.95 ± 3.52

### Microscale thermophoresis

**Fig. 3:**
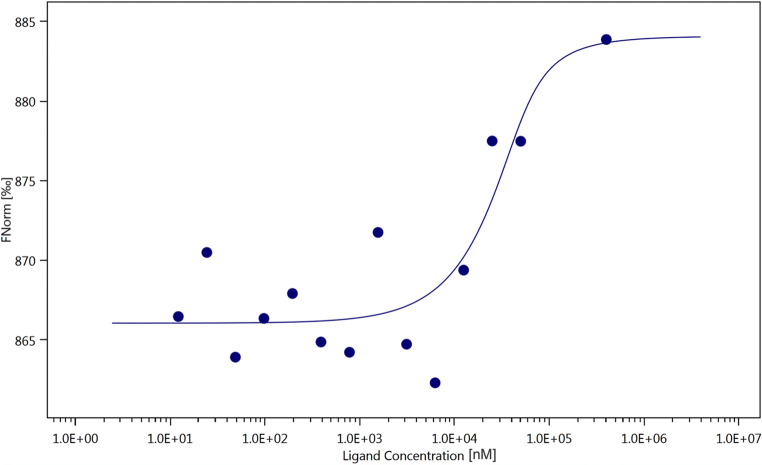
Binding of deschloro-chlorothricin to aurora kinase A as measured by MST. Experiment was performed with 60% LED power and 40% MST power. The curve shows the difference in the bound and unbound state of the aurora kinase A in presence of deschloro-chlorothricin. A fit was performed according to the law of mass action

The in vitro binding of DCCT to AURKA was verified with microscale thermophoresis. Different concentrations of DCCT ranging from 0.02 to 400 μM (1:1 dilution) were titrated against constant concentration of AURKA (400 nm). MST result showed a concentration-dependent fluorescence of AURKA against DCCT (Fig. 3). This suggest strong binding of the ligand DCCT to AURKA with a K_d_ value of 8.17 μM (standard error of regression: 3.41). This result confirmed the in silico results of binding between DCCT and AURKA.

### Cell cycle distribution

**Fig. 4:**
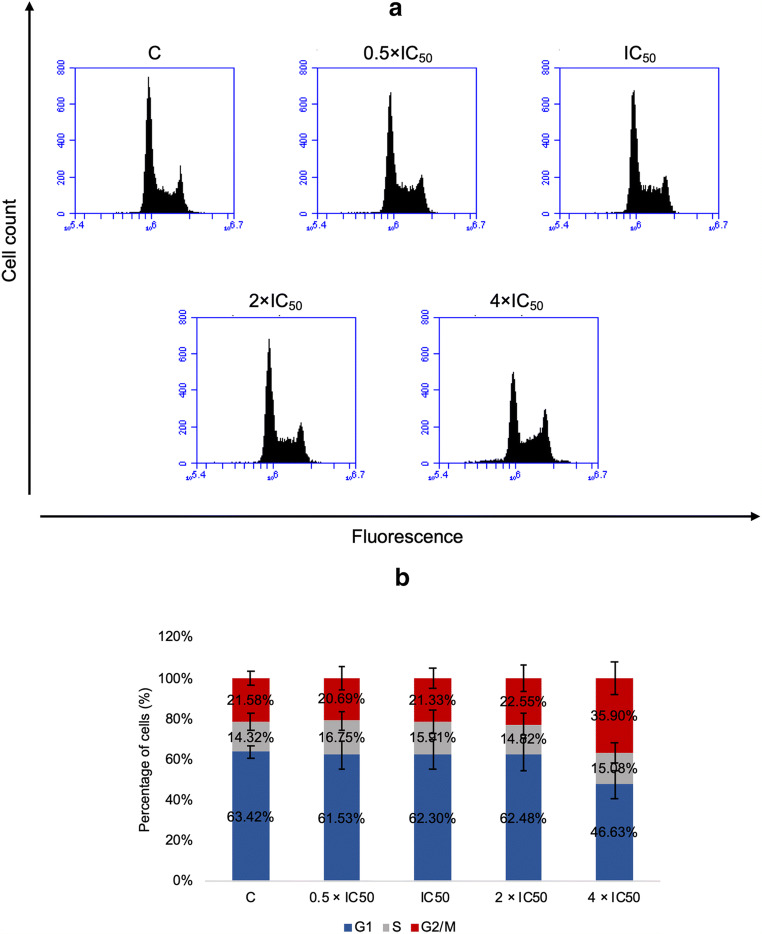
Induction of G2/M arrest by deschloro-chlorothricin. **a** MOLP-8 cells treated with DMSO or various concentrations of deschloro-chlorothricin stained with PI and analysed for DNA content by flow cytometry. **b** Quantitative analysis of the cell cycle distribution. The results represent the mean ± SD of three independent experiments

MOLP-8 cells were treated with ranging concentrations (0.5 × IC_50_, 1 × IC_50_, 2 × IC_50_, and 4 × IC_50_) of DCCT for 24 h, and then the effect of DCCT on cell cycle distribution were assessed. As shown in Fig. 4, a considerable increase in the cells in the G2/M phase of this cell line was observed if treated with 4 × IC_50_ of DCCT for 24 h.

### Influence of deschloro-chlorothricin (DCCT) on microtubules

**Fig. 5:**
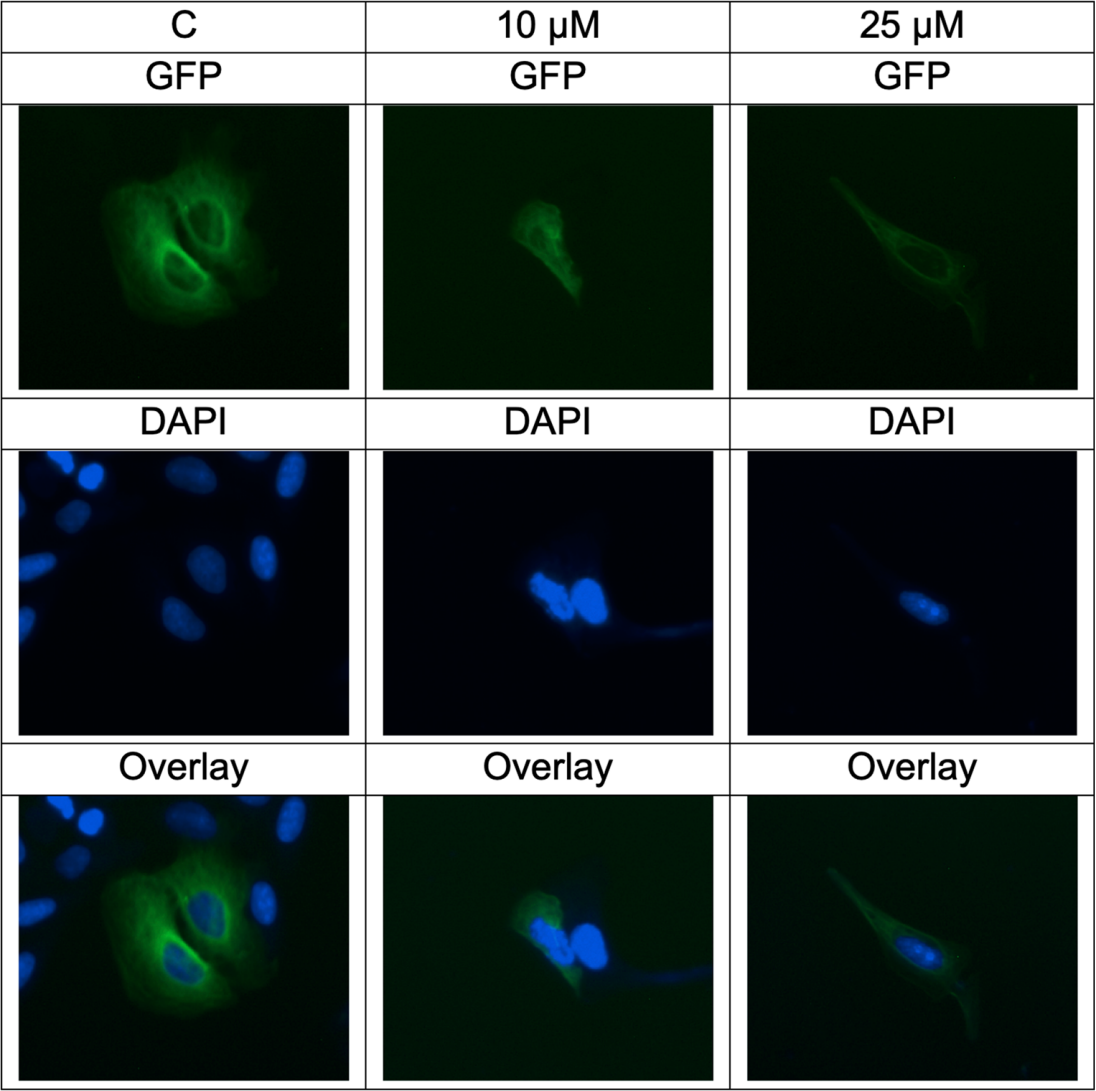
Deschloro-chlorothricin altered the morphology of the microtubule network in U2OS cells. Panels show the micrographs of U2OS cells treated for 24 h at 10 μM and 25 μM of deschloro-chlorothricin, respectively (Bar = 200 μM)

We treated U2OS cells, which express an α-tubulin-GFP fusion protein with various concentrations of DCCT to evaluate its influence on the cellular microtubule network. Figure 5 illustrates the dose-dependent effect of DCCT on the microtubule network. In non-treated cells, the microtubules continuously distributed throughout the cytoplasm and constituted an intracellular network apart from the nuclear region. The mass of the microtubule network in U2OS cells reduced in particular at the cell periphery if treated with DCCT. Their brightness and thickness decreased dose-dependently compared to non-treated cells, all pointing out the inhibitory effect of DCCT on microtubule formation (Fig. 5).

### Western blot analysis

**Fig. 6:**
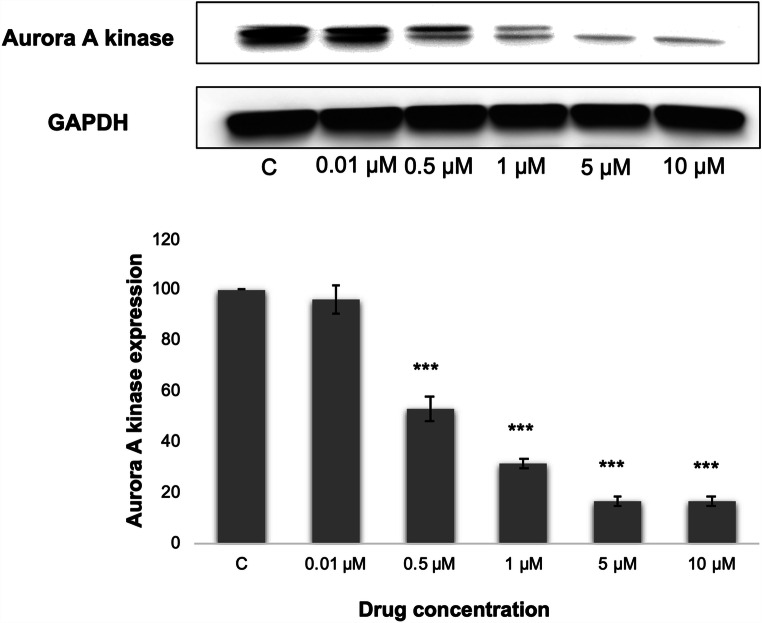
Western blot analysis of aurora kinase A in deschloro-chlorothricin-treated MOLP-8 cells. The cells were incubated with 0.01, 0.5, 1, 5, 10 μM concentrations of deschloro-chlorothricin for 24 h. Then, total protein was extracted and Western blotting was performed. The chart displays the change in the protein expression after normalization to GAPDH as mean ± SD for three independent experiments. Asterisks (***) indicated statistically significant downregulation by Student’s *t* test (*p* < 0.001) compared to DMSO-nontreated (control) cells

The impact of DCCT on the AURKA expression was evaluated by Western blot analysis. DCCT remarkably downregulated AURKA expression in a dose-dependent manner in DCCT-treated cells (Fig. 6).

## Discussion

### Virtual screening and molecular docking

In silico virtual screening and molecular docking were performed to predict probable ligands interacting with AURKA, which may provide potential drug candidates as promising AURKA inhibitors in cancer therapy. Nature as a unique resource and represents a great number of substances with potent bioactivities and presumably fewer side effects against cancer. Thus, screening a great number of natural compounds in the ZINC database targeting AURKs may result in the discovery of natural drug candidates and the construction of a natural library, which may provide lead structures or possibilities for the development of prospective (semi)synthetic drugs or combination interventions for the treatment of MM. Furthermore, computational approaches may enable the identification of repurposable drug candidates by saving time and reducing the costs of medications until reaching the market, all highlighting the prominence of in silico approaches [40–43].

DCCT was among the top 9 compounds with the highest binding affinity to AURKA based on in silico analyses, which was subsequently shown to be the only compound interacting with AURKA by microscale thermophoresis. Molecular docking analysis demonstrated its interactions with AURKA **(**Fig. 1b) as hydrogen bonds (Arg 137, Val 174 and Leu 178) and hydrophobic interactions (Leu 139, Lys 143, Phe 144, Val 147, Lys 162, Gln 177, Leu 194, Glu 211, Tyr 212, Ala 213, Leu 263, Ala 273, Asp 274, Phe 275, Gly 276) of the corresponding residues in the AURKA, which was quite similar to those of the AURKA inhibitor alisertib (hydrogen bonds at Arg 137 and Tyr 212, and hydrophobic interactions at Leu 139, Gly 140, Lys 141, Val 147, Ala 160, Lys 162, Leu 210, Glu 211, Ala 213, Pro 214, Leu 263, Ala 273, Asp 274) (Fig. 1c). Besides, an interaction of DCCT with vital amino acids in the binding pocket, enabled the understanding of the binding mode of the compounds, which may provide a model for prospective drug design for AURKA inhibitors. These amino acids are involved in the crystalized structure of the complex of AURKA and its inhibitor SAR156497 showing the interaction and include Val 147, Lys 162, Leu 178, Leu 194, Glu 211, Tyr 212, Ala 213, Leu 263, Ala 273, Asp 274 [25]. In addition to having a higher binding affinity to AURKA than alisertib, all findings pointed out the potential of DCCT as an novel AURKA inhibitor candidate.

### Cytotoxicity

A great number of natural compounds were reported to be active in vitro at concentrations of about 1 to 50 μM, and 15 μM was assigned as the target concentration generally used in most of the dose calculations [44]. On the other hand, the mean IC_50_ of 9 common chemotherapeutics was reported to be as low as 0.48 μM based on the simple analysis of data gained from the National Cancer Institute [44]. In the present study, we reduced the cut-off point to 10 μM. The top 9 compounds were tested at this concentration and only the compounds with an inhibitory effect ≥50% were regarded as highly cytotoxic against multiple myeloma cells. Two out of 9 compounds (ZINC000252515584 (DCCT) and ZINC000077262838) were identified as promising natural substances, among which only DCCT was further examined for the evaluation of detailed cytotoxicity on various MM cells and underlying molecular mechanisms due to interaction with AURKA.

DCCT displayed remarkable cytotoxicity on MM cell lines with extremely low IC_50_ values, which were even less than those of an AURKA inhibitor and clinical drug alisertib. To exemplify, a previous study indicated that alisertib inhibited cell viability on OMP-2 and RPMI-8226 cells with IC_50_ values of 4.37 and 10.32 μM, respectively [3], which were even higher than those of DCCT in our study, emphasizing the potential of the compound as promising natural drug candidate in MM interventions.

### Microscale thermophoresis

The outcomes of in silico virtual screening and molecular docking studies were validated by MST binding assay with purified in vitro. As assumed, the MST signals differed between the bound and unbound proteins, suggesting the interaction of the DCCT with AURKA.

### Cell cycle distribution

The expression and activity of all aurora kinases increase in mitosis. AURKA acts as a mitotic centrosomal kinase involved in chromosome maturation and regulation of G2/M phase transition [45]. During the proliferation of normal cells, AURKA is activated by phosphorylation during G2 to M phase transition in the cell cycle [18]. If the AURKA activity in tumor cells was disrupted, the accumulation of cells in G2/M phase and delayed mitotic entry were observed [46]. In another study, the inhibition of AURKA resulted in abrogation of G2/M cell cycle progression in MM cells [21]. Likewise, deschloro-chlorothricin induced G2/M arrest, as previously indicated for AURKA inhibitors in many reports [47–50].

### Influence of deschloro-chlorothricin (DCCT) on microtubules

The U2OS cells expressing α-tubulin-GFP were treated with DCCT to reveal the impact on the microtubule cytoskeleton by using digital inverted microscopy. Indeed, an unfavorable effect of DCCT was observed on microtubules, since the number of filaments as well as the intensity of tubulin staining reduced in dose-dependent manner.

The aurora kinases participate in cell cycle progression, mostly during the G2/M phases. AURKA is located on the poles of the mitotic spindle as well as the centrosomes and moves to centromeres during mitosis [13, 51, 52]. During mitosis, maintaining balanced chromosome segregation holds vital importance for the construction of a bipolar mitotic spindle. Because inadequacy in the spindle bipolar architecture may result in abnormal chromosome segregation and genetic instability in cancer cells. Microtubule-created forces have essential roles in the assembly of the mitotic bipolar spindle [53, 54]. These forces are associated with the dynamic microtubule properties and produced by the directional motion of motor proteins along microtubules [55]. Within the context of these findings, aurora A organizes centrosome assembly and stability, as well as nucleation and polymerization of centrosomal microtubules assigning a role in microtubule organization [56–59] and inhibitors may disrupt microtubule formation leading the repression of abnormal proliferation of cancerous cells. Therefore, DCCT inhibiting AURKA activity and thus, leading abnormal microtubule formation may be a promising drug candidate targeting AURKA in cancer therapy.

### Western blot analysis

AURKA enters the centrosome early in G2 and has been involved in the activation of CDK1/cyclin B on the centrosome [60]. Activated AURKA successively phosphorylating various centrosomal proteins, functions in centrosome maturation and mitotic spindle formation. The *AURKA* gene is usually overexpressed in cancer including MM [61, 62] and its amplification results in chromosome segregation anomalies related to malignant transformation both in vitro and in vivo [60, 63]. Moreover, the upregulation of AURKA inclined chemoresistance in breast cancer and ovarian cancer cells [16, 64]. In other studies, the suppression of AURKA expression enhanced paclitaxel-induced apoptosis in numerous cancer cells including kidney and breast cancer cell lines [65, 66].

Taken together, significant downregulation of AURKA by DCCT may provide additional strategies to combat MM, because it may either prohibit tumorigenesis through inhibiting chromosome segregation anomalies or improve the sensitivity of cancer cells to chemotherapeutics in the clinic. Further studies are required to emphasize the importance of the downregulation of aurora A in the chemosensitization of MM.

## Conclusion

In the present study, compound (5) (DCCT), a natural compound from the ZINC database, was identified as AURKA inhibitor based on in silico virtual screening and molecular docking studies, and the activity was experimentally validated in MM cells. DCCT was tested for the first time against several MM cells with a focus on determining its mechanism of action. Integration of computational and in vitro approaches enabled the experimental validation of in silico outcomes. DCCT remarkably inhibited the growth of MM cells, among which MOLP-8 cells were the most sensitive ones to the DCCT. These effects were related to the cell cycle arrest of the G2/M phase, the suppression of microtubule formation and the inhibition of AURKA activity and expression. The research not only renders a better understanding of how DCCT acts as antitumor agent on MM cells, but also provides information about the possible role of this drug in the treatment of MM. We introduced DCCT as a promising anti-cancer drug candidate worth further development.

## Abbreviations

AKI: Aurora kinase inhibitor
AURK: Aurora kinase
AURKA: Aurora A kinase
DAPI: 4′,6 diamidino-2-phenylindole
DCCT: Deschloro-chlorotricin
dlg: Docking log
FBS: Fetal bovine serum
MDR: Multi-drug resistance
MM: Multiple myeloma
MST: Microscale thermophoresis
NCI: National Cancer Institute
PDBQT: Protein data bank partial charge and atom type
PI: Propidium iodide
PIS: Penicillin (100 U/ml)-streptomycin (100 μg/ml)
PBS: Phosphate buffered saline
*pKi*: The predicted inhibition constant
SEER: The Surveillance, Epidemiology, and End Results
TBST: Tris-buffered saline Tween 20
VMD: Visual molecular dynamics
