# Correction to: Identification and characterization of deschloro-chlorothricin obtained from a large natural product library targeting aurora A kinase in multiple myeloma

**DOI:** 10.1007/s10637-021-01193-4

**Published:** 2021-10-27

**Authors:** Nadire Özenver, Sara Abdelfatah, Anette Klinger, Edmond Fleischer, Thomas Efferth

**Affiliations:** 1 Department of Pharmacognosy, Faculty of Pharmacy, Hacettepe University, 06100 Ankara, Turkey; 2 Department of Pharmaceutical Biology, Institute of Pharmaceutical and Biomedical Sciences, Johannes Gutenberg University, Staudinger Weg 5, 55128 Mainz, Germany; 3 MicroCombiChem GmbH, 6520 Wiesbaden, Germany

## Correction to: Invest New Drugs (2021) 39:348–361 https://doi.org/10.1007/s10637-020–01012-2

The article **Identification and characterization of deschloro-chlorothricin obtained from a large natural product library targeting aurora A kinase in multiple myeloma** was originally published electronically on the publisher’s internet portal on 25 September 2020 without open access. After publication in volume [39], issue [2], pages [348–361] the author decided to opt for Open Choice and to make the article an Open Access publication. Therefore, the copyright of the article has been changed to © The Author(s) 2021 and the article is forthwith distributed under a Creative Commons Attribution 4.0 International License, which permits use, sharing, adaptation, distribution and reproduction in any medium or format, as long as you give appropriate credit to the original author(s) and the source, provide a link to the Creative Commons licence, and indicate if changes were made. The images or other third party material in this article are included in the article’s Creative Commons licence, unless indicated otherwise in a credit line to the material. If material is not included in the article’s Creative Commons licence and your intended use is not permitted by statutory regulation or exceeds the permitted use, you will need to obtain permission directly from the copyright holder. To view a copy of this licence, visit. http://creativecommons.org/licenses/by/4.0. Open access funding enabled and organized by Projekt DEAL.

The original article has been corrected.

